# Graphene Quantum Dots from Natural Carbon Sources for Drug and Gene Delivery in Cancer Treatment

**DOI:** 10.3390/ijms251910539

**Published:** 2024-09-30

**Authors:** Henrry M. Osorio, Fabián Castillo-Solís, Selena Y. Barragán, Cristina Rodríguez-Pólit, Rebeca Gonzalez-Pastor

**Affiliations:** 1 Departamento de Física, Escuela Politécnica Nacional, Av. Ladrón de Guevara E11-253, Quito 170525, Ecuador; henrry.osorio@epn.edu.ec (H.M.O.); selena.barragan-maldonado@uni-wuerzburg.de (S.Y.B.); 2 Centro de Investigación Biomédica (CENBIO), Facultad de Ciencias de la Salud Eugenio Espejo, Universidad UTE, Quito 170527, Ecuador; fabianc.castillo@ute.edu.ec (F.C.-S.); crodriguez@inspi.gob.ec (C.R.-P.); 3 Escuela de Salud Pública, Universidad San Francisco de Quito USFQ, Quito 170527, Ecuador; 4 Centro de Referencia Nacional de Genómica, Secuenciación y Bioinformática, Instituto Nacional de Investigación en Salud Pública “Leopoldo Izquieta Pérez”, Quito 170403, Ecuador

**Keywords:** sustainable carbon, graphene quantum dots, natural carbon sources, nanomedicine, biocompatible nanocarriers, photoluminescence, cancer therapy, drug and gene delivery, targeted delivery systems, combinatorial strategies

## Abstract

Cancer therapy is constantly evolving, with a growing emphasis on targeted and efficient treatment options. In this context, graphene quantum dots (GQDs) have emerged as promising agents for precise drug and gene delivery due to their unique attributes, such as high surface area, photoluminescence, up-conversion photoluminescence, and biocompatibility. GQDs can damage cancer cells and exhibit intrinsic photothermal conversion and singlet oxygen generation efficiency under specific light irradiation, enhancing their effectiveness. They serve as direct therapeutic agents and versatile drug delivery platforms capable of being easily functionalized with various targeting molecules and therapeutic agents. However, challenges such as achieving uniform size and morphology, precise bandgap engineering, and scalability, along with minimizing cytotoxicity and the environmental impact of their production, must be addressed. Additionally, there is a need for a more comprehensive understanding of cellular mechanisms and drug release processes, as well as improved purification methods. Integrating GQDs into existing drug delivery systems enhances the efficacy of traditional treatments, offering more efficient and less invasive options for cancer patients. This review highlights the transformative potential of GQDs in cancer therapy while acknowledging the challenges that researchers must overcome for broader application.

## 1. Introduction

Cancer remains a primary global health concern and a leading cause of mortality worldwide, with 20 million new cases and 9.7 million deaths reported in 2022 [1]. This disease not only represents a significant challenge to public health but also imposes a substantial socioeconomic burden due to its extensive associated costs [2]. Conventional therapies, including surgery, radiotherapy, and chemotherapy, often fall short due to the complex and heterogeneous nature of tumors, resulting in significant collateral damage to healthy cells. Additional therapeutic strategies have been developed to address these limitations, including hormonal therapy, targeted therapy, immunotherapy, and gene and cell therapy [3]. Gene and cell therapies, in particular, have demonstrated potential in preclinical studies by improving the precision and selectivity of treatments directed at cancer cells [4]. However, the clinical application of these therapies is hindered by challenges such as the inefficient delivery and release of therapeutic agents within the tumor microenvironment [5,6]. Recent advancements in cancer treatment have transformed the landscape, driven by a deeper understanding of cancer’s multifaceted nature, including genomic aberrations, dysregulated cellular pathways, and the tumor microenvironment [7,8]. This insight has paved the way for the development of innovative treatments targeting specific genetic alterations and other molecular features of cancer cells [9]. Approaches such as oncolytic viruses, genetic modification of cancer and immune cells, and siRNA technology have led to numerous clinical trials, some of which are in late-stage development [10]. Nevertheless, the implementation of these advanced therapies poses significant financial challenges, emphasizing the need for cost-effective solutions and equitable access to care, in line with the objectives of SDG 3 to ensure healthy lives and promote well-being for all at all ages [11].

In recent decades, nanomedicine has experienced accelerated growth in the design and synthesis of nanoscale vectors applied to cancer treatment [12,13]. These nanovectors include liposomes, micelles, dendrimers, metal nanoparticles, and carbon-based nanoparticles [14]. Nanoparticles can typically range from 1 to 100 nm, where quantum nature dominates over bulk properties due to the nanoscale dimensions. This results in a high surface-to-volume ratio and quantum effect such as the quantum confinement [15,16], which alters optical properties, including photoemission in semiconductor and carbon-based materials. Thanks to their size, nanoparticles can cross physiological barriers and interact with biomolecules within cells and organs at multiple levels. Also, these nanoscale vectors can remain at the tumor site given their enhanced permeability and retention (EPR) effect [17,18]. Furthermore, their surface chemistry can be tailored to support the attachment of therapeutic agents, including genes, hormones, and drugs. This adaptability also allows for the integration of features like reduced uptake by the reticuloendothelial system and controlled drug delivery, which are essential for designing more specific and effective therapies [19]. Thus, nanoparticles have been utilized to develop new treatments based on their physical properties and to enhance conventional cancer therapies, also serving as multifunctional platforms, enabling theranostic [20] and combined therapies [21].

In this context, carbon is one of the most abundant elements and forms multiple structures [22], including graphite, fullerenes [23], and graphene [24]. Graphene in particular has attracted considerable interest in different areas due to its exceptional thermal, mechanical, optical, and electronic properties, such as high intrinsic mobility, a null effective mass, and a zero-bandgap energy [25]. Furthermore, as observed for other nanoparticles, graphene exhibits quantum confinement effects that alter its electronic properties, resulting in a non-zero-bandgap and tunable photoluminescence depending on its size [26]. These atomically flat but zero-dimensional materials based on graphene are known as graphene quantum dots (GQDs). Undoubtedly, this exclusive structure offers unique properties that have been extensively studied in various areas and novel applications such as energy conversion and storage, catalysis, flexible devices, sensing, and display [27,28], as well as in biomedical applications like diagnostics, treatment, bioimaging, and biosensing [20,29,30,31,32,33].

GQDs offer several advantages that make them highly suitable for cancer therapy. Their high surface-to-volume ratio and versatile structure allow for different types of surface functionalization, making GQDs essential platforms for the anchoring and transporting of chemotherapeutic drugs, genes for gene and cell therapy [34], photosensitizers for photodynamic therapy (PDT) [35], heating agents under NIR light for photothermal therapy (PTT) [36], and different cancer-targeting ligands [37]. Their inherent carbon structure and specialized surface functionalization ensure high biocompatibility, low toxicity, and biodegradability, further enhancing their suitability for biomedical applications [38,39]. The biodegradable nature of GQDs aligns with SDG 12, as it underscores the importance of responsible production and consumption, reducing the environmental impact of nanomaterial-based cancer treatments [11]. Furthermore, their photoluminescence enables precise and controlled monitoring of the transported therapeutic agents [40]. These properties collectively contribute to the development of combined therapies using GQDs, leveraging their synergistic effects to enhance therapeutic efficiency.

Despite their advantageous properties, achieving precise properties is challenging due to the sensitivity of these properties to synthesis conditions. Various methods exist for the synthesis of GQDs, each with its own advantages and limitations [41,42]. To unlock the full potential of GQDs for diverse applications, it is crucial to develop novel synthesis strategies that address existing challenges, such as achieving uniform size and morphology, precise bandgap engineering, scalability, minimizing contaminants, and mitigating the environmental impact involving the synthesis reagents and carbon sources [43,44,45]. Such approaches are essential to advancing sustainable nanotechnology solutions, in line with SDG 9 (Industry, Innovation, and Infrastructure) [11].

**Figure 1:**
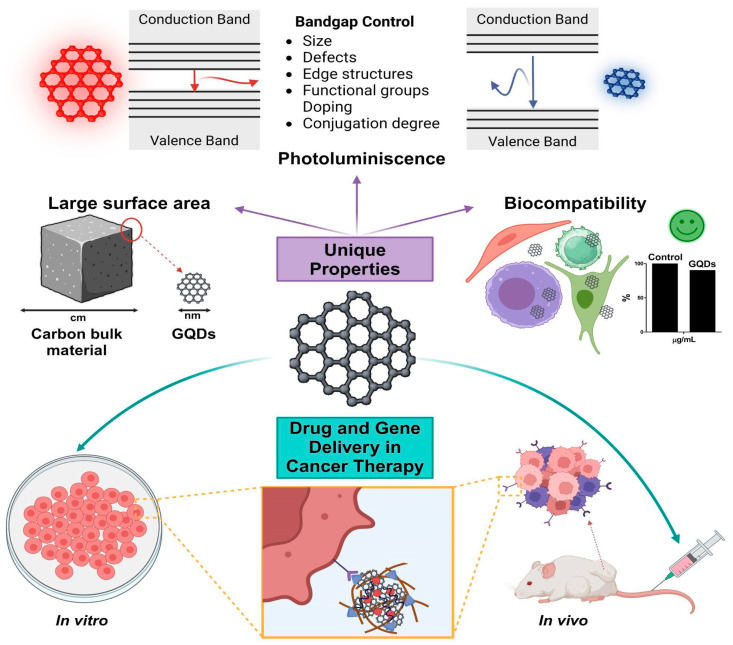
Properties and applications of GQDs in cancer therapy. High surface area enables efficient drug loading and interaction with biological molecules; photoluminescence allows tracking and can be used to trigger drug release and to induce cytotoxic effects in cancer cells; biocompatibility ensures safety for use in biological environments; and drug and gene delivery allow targeted transport and controlled release of chemotherapeutics and expression of therapeutic genes in cancer cells. Figure was created using Biorender.com (accessed on 1 June 2024).

Prompted by the current landscape, this review explores the recent advances in developing nano-formulations based on GQDs for cancer treatment. We discuss novel methodologies for obtaining GQDs and the unique properties that make them suitable for cancer therapy. Special emphasis is placed on the use of GQDs for drug and gene delivery for cancer treatment, examining their functionalization, efficiency, biocompatibility, and toxicity. Figure 1 highlights the main properties related to the applications of GQDs in cancer therapy.

## 2. Production of GQDs from Natural Carbon Sources

### 2.1. Methods of GQDs Production from Natural Carbon Sources

The preparation of GQDs generally falls into two categories: top-down and bottom-up methodologies. Top-down approaches involve utilizing large carbon materials that have large sp^2^ carbon domains. These materials are then subjected to processes that reduce their size and produce GQDs by cutting down the structure and obtaining small pieces of material, while preserving the sp^2^ structure in the inner part. On the other hand, bottom-up methodologies utilize carbon-based small molecules to fuse them, constructing the sp^2^ domains.

Graphite represents the natural material to fabricate GQDs through top-down methodologies. Graphite can be cut down to obtain GQDs using electrochemical and liquid-phase exfoliation techniques. In the electrochemical approach, GQDs are obtained by electrolysis using a two-electrode configuration. A graphite electrode is used as a working electrode together with an appropriate counter electrode, generally platinum. Both electrodes are immersed in an electrolyte solution, which is often used to control the functionalization of GQDs. Applying a voltage between electrodes, the graphene electrode is exfoliated, obtaining pieces of different sizes. Then, the solution is either centrifuged or filtrated to select the smallest pieces, the GQDs. Finally, the solution is subjected to dialysis or distillation to purify the GQDs [46]. In the liquid-phase exfoliation technique, graphite powder is dispersed in a suitable solvent and is complemented by ultrasonic agitation to cut down the layered structure. During ultrasonication, shear forces and cavitation induce exfoliation of the layered structure into small particles. The success of the methodology depends on the solvent used. Solvents with a surface tension around 40 mJ·m^−2^ are the best solvents, such as N-methyl-2-pyrrolidone (NMP) and N,N-dimethylformamide (DMF). At the end of the process, the resulting dispersion has to be processed to separate the GQDs from larger particles by centrifugation, filtration, and/or dialysis [47,48].

While electrochemical and liquid-phase exfoliation techniques applied to graphite allow the production of GQDs, the largest amount of exfoliated material is graphene, monolayered or multilayered, and non-exfoliated graphite. This results from exfoliation techniques overcoming the van der Waals attractions between adjacent layers of graphene with graphite, but the rupture of C-C bonds cannot be achieved. The higher the sp^2^ content in the structure, the greater the crystallinity and the fewer the defects, resulting in larger exfoliated particles. To obtain GQDs through top-down methodologies, cutting occurs at the defect sites, and the more defects, the more cutting sites. Therefore, if defects are introduced into the graphite or graphene structure before the exfoliation process, it will be easy to cut down the structure to obtain GQDs. In this context, another technique used to obtain GQDs is the hydrothermal/solvothermal technique. This technique utilizes graphene oxide (GO), which possesses a similar sp^2^ structure to graphene but with a larger amount of defect sites. GO can be obtained from graphite using the Hummers and modified Hummers methods [49,50,51]. The hydrothermal/solvothermal method consists of subjecting GO to high temperatures and pressures using an autoclave, which cuts down the structure through the defect sites, forming GQDs [52,53,54]. The obtained GQDs particle size can be modified by adjusting the hydrothermal/solvothermal temperature. An increase in temperature reduces the particle size of GQDs [55]. Additionally, reactants can be included in the autoclave to control the functionalization of GQDs. This methodology has also been used to cut down other graphene-based nanomaterials, such as carbon nanotubes [56] and fullerenes [57], to obtain GQDs. Another routine to cut down GO to obtain GQDs is using chemical methods. Here, strong acids or oxidizing agents fragment GO through their functional groups. The sizes of the obtained GQDs can be modified by varying the reaction temperature [58].

For their part, bottom-up methods for the preparation of GQDs utilize carbon-based small molecules such as amino acids, carbohydrates, citric acid, sucrose, glucose, pyrene, and trisodium citrate, among others, as carbon sources [59]. Here, molecules are fused to generate GQDs. In the first instance, the temperature of these molecules can be elevated until the melting point, which leads to the fusion of the molecules forming the GQDs. The increase in the temperature can be achieved using carbonization pyrolysis, hydrothermal/solvothermal method, or by explosion [60,61,62,63]. Controlling the reaction parameters leads to controlling the size of the GQDs [64]. Also, carbon-based molecules are combined with reagents in order to functionalize the GQDs [65]. Solution chemistry [66,67] and microwave irradiation [68] also appear as important routes to fuse carbon-based molecules to produce GQDs. The versatility of bottom-up methods makes them suitable for controlling the size of GQDs, but requires the use of ligands and several purification steps, especially at the end of each reaction stage.

### 2.2. Novel Approaches and Innovations in GQDs Production

While standard methods described previously allow the preparation of GQDs at the laboratory level, there are several challenges that must be overcome before GQDs can be implemented in the market, especially for cancer treatment. Both top-down and bottom-up methodologies use toxic reagents, strong acids, or potent oxidizing agents, which must be treated after the production process. Also, it is important to consider the reagents used in the pre-fabrication processes, such as the production of GO. Top-down methodologies align more closely with mass production for market implementation, but often result in GQDs with poor control over size and morphology, resulting in a wide variation in their properties. In contrast, bottom-up methodologies offer better control over the size and morphology of GQDs. However, these methods produce smaller quantities of GQDs, which limits their market applicability. We present the improvements developed in the last years to overcome the challenges associated with GQDs’ production.

Related to the electrochemical exfoliation to cut down graphene-based materials into GQDs, this methodology requires a solid graphite working electrode in principle. However, materials with large sp^2^ carbon domains that are easier to cut down, such as graphene or GO flakes, could be used if pressed or drop-casted onto a conductive material to form an electrode [69,70]. In addition, pre-treatment processes applied to the starting material can be implemented to create defects in the morphology, increasing the cutting sites and, consequently, the efficiency of the GQDs production. For example, pre-heating of graphite rods at 1050 °C before electrochemical exfoliation produces defects on the surface of graphite, which facilitates the cut-down and production of GQDs [71]. In the same way, graphite can also be treated before applying liquid-phase exfoliation techniques to create defects that facilitate the cutting down of the structure. Thus, an innovative pre-treatment that consists of immersing the precursor material into liquid nitrogen up to 10 h before the liquid-phase exfoliation has been proposed [72]. Also, defects can be introduced using chemical reagents. For example, acid treatment applied to GO can be used to facilitate the production of GQDs in hydrothermal/solvothermal methods [73]. In parallel, there has been growing attention to promoting better control of the properties of the obtained GQDs using top-down methods. Thus, it has been demonstrated that surface passivation can be controlled by incorporating glucose in the electrolytic solution and applying microplasmas during the electrochemical exfoliation process. This control allows the formation of excitation-independent GQDs using high surface passivation by oxygen [74].

Researchers have also proposed using innovative reagents that promote an efficient and/or eco-friendly cut-down of graphite structures. In this way, ionic liquid 1-butyl-3-methylimidazolium hexafluorophosphate (BMIMPF_6_) has been used satisfactorily as electrolyte to cut down self-supported foam-like 3D graphene to obtain GQDs using electrochemical exfoliation [75]. Likewise, supercritical CO_2_ has proved to be an efficient solvent for obtaining GQDs using liquid-phase graphite exfoliation [76,77]. In the same way, it has been demonstrated that a mixture of ethanol/water can also be used as an eco-friendly solvent to obtain GQDs in the liquid-phase exfoliation of graphene [78]. In the case of using chemical methods to cut down graphene structure, the use of green oxidants to replace strong acids has been extensively studied. For example, H_2_O_2_ has been employed as an oxidant to produce GQDs from graphene, obtaining only H_2_O and GQDs as products [79]. In addition, an oxidant combined with the sonochemical strategy and microwave radiation has been used satisfactorily to increase the efficiency of producing GQDs [80].

On the other hand, bottom-up methodologies can be improved by implementing processes that increase the quantity of produced GQDs, involve lower reaction temperatures, and perform an efficient route to achieve atomic-level accuracy. It has been probed that the use of microwave irradiation heating induces the decomposition of molecules, facilitating their assembly in the pyrolysis and hydrothermal/solvothermal methods [68,81,82]. In addition, novel solution chemistry processes, e.g., catalytic solution chemistry, can be implemented in order to induce chemical changes in molecules to accomplish their fusion to obtain GQDs [59]. Also, the synthesis and use of templates further complement the bottom-up methodologies, especially in order to obtain GQDs with high accuracy in their morphology. Thus, polycyclic aromatic hydrocarbons (PAHs), hexaborylated [6]cyclo-m-phenylene, and polyethylene glycol have been satisfactorily used to obtain efficient templates in bottom-up synthesis of GQDs [83,84,85].

**Figure 2:**
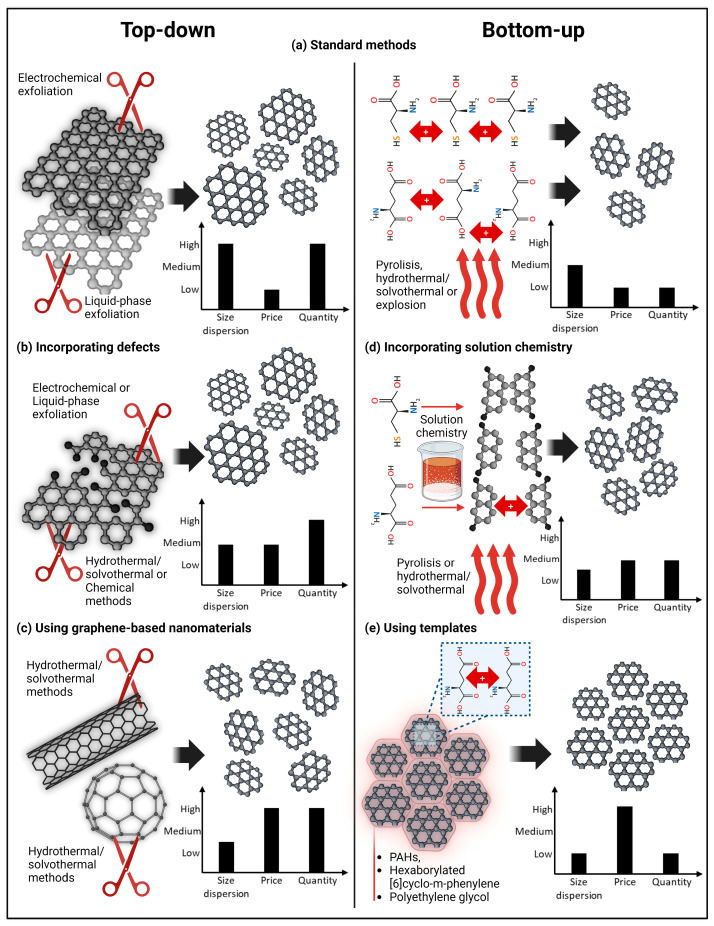
Illustration of standard methods of GQDs production and their innovations. (**a**) Standard methods, top-down and bottom-up approaches; (**b**) introducing defects at the graphene sp^2^ structure facilitates cutting the structure to obtain GQDs; (**c**) graphene-based nanomaterials, such as carbon nanotubes and fullerenes can be utilized to obtain GQDs by means of the top-down methods; (**d**) solution chemistry processes induce chemical changes in molecules to facilitate their fusion to produce GQDs; (**e**) use of templates to produce GQDs improves the control in their sizes and morphologies. Insets show characteristics of each method. For comparison, requirements to implement GQDs in market are: low size dispersion, low price, and high quantity of produced material. Figure was created using Biorender.com (accessed on 1 June 2024).

It is also worth mentioning that bottom-up methods have also been exploited to produce graphene or graphitic structures from molecules such as PAHs or cysteine [86,87,88,89]. These artificial graphene and graphite have also been demonstrated to be suitable for cutting down using exfoliation or hydrothermal/solvothermal methods to obtain GQDs with well-defined morphology, diameter, and thickness [89,90]. Figure 2 illustrates the standard methods of GQDs production and their innovations.

### 2.3. Advantages and Limitations of Natural Sources

Many carbon materials have been utilized in the production of GQDs. Since the characteristics of the resulting product, and consequently, its suitability for future applications, can vary depending on the process and precursors, the selection of the source is equally as important as the selection of the fabrication method [91]. As discussed above, top-down methods are well-suited for the mass production of GQDs, with carbon materials containing large sp² carbon domains being ideal sources for GQD preparation. However, this strategy requires particular instruments and equipment, such as high-pressure and high-temperature equipment, resulting in low yields, limited production scalability, and increased production cost. Additionally, while using graphene, GO, carbon nanotubes, or fullerenes as starting materials increases the performance in the production of GQDs, producing these materials before the GQDs preparation also represents an increase in the production cost. To reduce production costs, carbon-based abundant natural resources have been proposed as sources for GQDs production, applying especially but not limited to top-down methodologies.

Coal and coke consist of small graphite-like clusters arranged in disordered structures with varying degrees of graphitization. Coke, produced through high-temperature thermal treatment of anthracite coal, has a higher degree of graphitization compared to coal. Due to their disorder and defects, both coal and coke are easier to cut down into GQDs than graphite [92]. Chemical methods are commonly employed to achieve this transformation. As the degree of graphitization increases, as seen with anthracite, the size of the produced GQDs shows a wider dispersion, ranging from 1 to 7 nm. Conversely, a lower degree of graphitization, such as in bituminous coal, results in a narrower GQD size distribution, from 1 to 3 nm [93]. However, using coke with a higher degree of graphitization leads to a significant increase in the size dispersion of the produced GQDs, ranging from 40 to 100 nm [94]. These findings suggest that controlling the graphitization degree in the source material is crucial for achieving better control over the size of the GQDs produced. Because of the compromise between the graphitization degree and the dispersion in the size of the GQDs, it is important to use a natural source with a convenient degree of graphitization that is not too high or too low. Considering that chemical exfoliation uses strong acids or oxidizing agents, other top-down procedures should be implemented to obtain GQDs from coal and coke. In this context, electrochemical exfoliation has also been used to satisfactorily obtain GQDs from coke. Remarkably, the right electrolyte selection conduces to the production of GQDs with low size dispersion [95]. 

Another source to produce GQDs is biomass-carbonaceous waste. Biomass-based GQDs and other carbon-based quantum dots have gained attention due to their biocompatibility and the abundance of biomass as a renewable and sustainable resource [96]. Biomass sources such as plants and animal derivatives, as well as municipal waste, can be used to prepare GQDs [46,97]. In this case, GQDs can be obtained using both top-down and bottom-up methodologies. To use a top-down strategy, biomass is carbonized to promote the formation of large sp^2^ carbon domains that are subsequently cut down [98]. On the other hand, to use a bottom-up strategy, biomass is treated to separate it into small molecules that can be fussed using pyrolysis, chemical, or hydrothermal/solvothermal routes to obtain GQDs [99,100]. Lastly, amino acids, the building blocks of proteins, have been employed as natural sources for GQD preparation via pyrolysis, a bottom-up approach [101,102]. The use of microwaves for uniform heating during this process enhances size control of the GQDs and provides the added benefits of good biocompatibility and low toxicity [103]. It has been demonstrated that byproducts of the food supply chain can be reused to produce GQDs. These byproducts contain different natural carbon-based molecules which make them a diverse source for GQDs production. Thus, natural carbon sources as wastes derived from vegetables, fruits, livestock, and the food industry contribute to the development of a sustainable GQDs production without contending with food suppliers and without use of any potential toxic chemical reagents [104].

Table 1 presents a compilation of carbon sources, fabrication methods, functionalization, size/thickness, and applications of the GQDs obtained in previous studies.

## 3. Unique Properties of GQDs for Cancer Therapy

### 3.1. Morphological, Optical and Electronic Properties

GQDs are composed of sp^2^-hybridized carbon atoms arranged in a hexagonal lattice, like graphene. This configuration allows for the delocalization of π electrons along the carbon–carbon bonds, forming a conjugated π system [24,25]. In graphene, this electron delocalization provides unique electronic properties, such as high electron mobility and optical transparency [116,117]. The delocalization of electrons within the graphene core of GQDs plays a key role in their unique properties, with quantum confinement effects becoming prominent due to their small size [62,118,119,120]. While quantum confinement leads to discrete energy levels for electrons, the bandgap depends on the final structure of GQDs, which varies based on the synthesis method [121,122] and conditions [27]. This variability leads to differences in size [123,124], shape [125], defects [126], edge structures [127,128,129], functional groups [130,131,132,133], doping [134,135,136,137,138,139,140,141], and conjugation degree [142,143,144]. Theoretical calculations can study GQDs with specific structures [145,146] precise production of identical GQDs with identical morphology, and in consequence, with identical properties, remains a big challenge that needs to be addressed [31,76,147,148].

GQDs exhibit strong absorption in the ultraviolet region, showing the formation of a broad absorption peak, which is attributed to π-π* transitions within the sp^2^-hybridized carbon structure [52,149]. As the size of the GQDs decreases, the absorption peak tends to blue-shift due to quantum confinement effects [58,150]. Functionalization and doping can also affect the absorption properties of GQDs. In this context, GQDs exhibit a shoulder peak extended to visible and NIR regions in the absorption spectra attributable to n–π* transitions in C=O bonds promoted by introducing oxygen groups [151,152]. In addition, GQDs functionalized with electron-donating groups may exhibit red-shifted absorption peaks, while those functionalized with electron-withdrawing groups may show blue-shifted peaks [57,153,154,155,156,157]. As mentioned before and similar to the absorption changes, quantum confinement causes size-dependent photoluminescence, where smaller GQDs exhibit a larger bandgap, and thus blue-shifted emission [58,150,158,159]. Additionally, their photoluminescence is often excitation wavelength-dependent, with emission characteristics varying according to the excitation light wavelength [160,161,162] and the efficiency of light emission (quantum yield) is typically high [163,164,165,166]. Excitation dependent emission from GQDs is one of the key properties that represents a substantial body of research. The main proposal to explain this behavior is based on the idea of selective excitation of different sizes corresponding to different dominant bandgaps [52,90,143]. However, recent studies indicate that excitation dependent emission depends on surface states, which involve energy transfer or charge transfer mechanisms [167,168]. Simultaneously, other studies have observed excitation independent photoluminescence emission from GQDs [169,170,171,172]. The photoluminescent properties of GQDs are of significant interest due to their potential applications in various fields, including bioimaging, sensing, and optoelectronics [26,27,28,29,30,31,173].

While GQDs are widely recognized for their photoluminescence [121], they also exhibit other crucial properties for cancer therapy. Conjugated π system and discrete energy levels formed through quantum confinement allow the use of GQDs for PTT and PDT. In these therapies, GQDs are delivered to cancer cells and then irradiated. In the case of PTT, cancer cells are irradiated with NIR light. GQDs absorb NIR radiation, which is efficiently converted into heat, producing localized cancer cell death while minimizing damage to healthy tissue [36,174]. Their high photothermal conversion is attributed to substantial optical absorption in the NIR directly related to the degree of π conjugation and low-energy vibrational modes. When π conjugation is disrupted, for example through oxidation of the structure, the optical absorption decreases in the visible and infrared [175]. Thermodynamic vibrations, nonradiative relaxation, and plasmonic localized heating are the three different mechanisms that constitute the photothermal conversion process at GQDs [176]. Thus, as long as a high π conjugated degree is maintained, top-down and bottom-up-synthesized GQDs can be successfully used as PTT agents [174]. In the case of PDT, cancer cells are irradiated with visible light. GQDs absorb visible radiation, and this energy is used to produce reactive oxygen species (ROS), specifically singlet oxygen (^1^O_2_), which induce cytotoxicity in target cells [177,178,179]. GQDs present higher ^1^O_2_ generation yield compared to conventional photosensitizers as protoporphyrin IX due to the multi-state sensitization mechanism (MSS) promoted by GQDs. In conventional PDT, when PDT agents absorb energy, these go to their respective excited singlet state (S1). Subsequently, an intersystem crossing transition is produced and agents go to their excited triplet state (T1). Finally, agents return to their respective ground states (S0) by transferring energy to molecular oxygen in triplet ground state (^3^O_2_). As a result, ^1^O_2_ is generated. In MSS, GQDs absorb energy and present two times ^1^O_2_ generation yield. First, during the intersystem crossing transition in GQDs (S1 → T1); and second, when GQDs return to their respective ground states (T1 → S0). This MMS is promoted because GQDs present an energy gap between S1 and T1 larger than the formation energy of ^1^O_2_, contrary to conventional photosensitizers [121]. This property has been observed specially in GQDs with tunable oxygen functional groups prepared by electrochemical exfoliation or hydrothermal method varying the alkali hydroxide concentration in the used solution [177,179].

While it has been probed that GQDs irradiated with visible light generate ROS and kill cells by causing oxidative stress, when they are not irradiated they can present high antioxidant activity. Both the total oxygen content and the type of oxygen groups contribute to the antioxidant activity of GQDs. The principal mechanism for free radical scavenging is attributed to the hydrogen donor from the surface produced specially by hydroxy and carbonyl moieties introduced when GQDs are prepared under NaOH or H_2_O_2_ electrolysis environment [180,181,182].

The conjugated π system promoted by the hexagonal structure of GQDs facilitates the self-assembly of different compounds onto the surface of the GQDs through non-covalent π-π interactions. Additionally, GQDs possess an extremely high surface-to-volume ratio due to their small size and planar structure [183,184]. This combination of characteristics is highly advantageous for applications requiring extensive interaction sites and increased reactivity, such as in catalysis, sensing, and energy storage [185,186,187,188,189,190,191]. In cancer treatment, GQDs serve as effective carriers for drug and gene delivery [34,37,192]. In addition, the heat generated by GQDs under light irradiation can trigger the release of the drug at the target site, enhancing delivery efficiency [193,194,195]. This versatility positions GQDs as a powerful platform for developing combinatorial cancer therapies that may achieve additive or synergistic therapeutic effects, improving treatment efficacy towards malignant tissue while reducing toxicity over healthy cells [36,196,197]. Additionally, GQDs’ photoluminescence enables their use as imaging agents in cancer diagnosis [198], in theranostics that combine diagnosis with treatment [20,192,199], and for real-time monitoring of therapeutic agent delivery, verifying targeting accuracy and assessing treatment effectiveness [177,200,201,202,203].

A relatively new observed property of GQDs promoted by the quantum confinement is the up-conversion photoluminescence. This is referred to as a process where low-energy photons (e.g., infrared light) are absorbed and converted into higher-energy photons (e.g., visible or ultraviolet light). This process involves multiple photon absorption events followed by energy transfer processes within the GQDs [204,205,206]. Applications of up-conversion photoluminescence in GQDs include bioimaging, where infrared light penetrates deeper into tissues and is absorbed by the GQDs, which then convert it into visible or ultraviolet light for capturing high-resolution images [207,208]. These properties make GQDs with up-conversion capabilities attractive for use in various imaging and sensing applications [209,210,211].

### 3.2. Drug and Gene Loading and Release Properties

In cancer therapy, GQDs have been used to transport drugs, genes, and other molecules with therapeutic activity [34]. Their extensive surface area, variable number of graphene layers, the presence of π electrons, and the ability to be doped with heteroatoms facilitate the formation of plentiful polar groups by providing abundant reactive sites, influencing their overall chemical reactivity and interaction with other molecules or materials [34,40]. These polar groups facilitate the binding of GQDs to aromatic molecules through various interactions, mainly π-π interactions, hydrogen bridges, and electrostatic interactions [212,213,214]. These characteristics enable the incorporation of hydrophobic chemotherapeutics on their surface, the production of more stable complexes, and increased solubility in various solvents [215]. The sp^2^ nature of the surface [216] and its double face allow GQDs to incorporate molecules forming a sandwich-like structure through π-π interactions [217]. Under physiological pH conditions (7.4), the hydroxyl groups on the GQDs ionize to form oxide ions (-O), which improves the loading capacity of molecules such as doxorubicin (DOX), methotrexate (MTX), and mitoxantrone (MTN) [113,121,217,218]. Furthermore, these interactions can be stabilized by increasing the presence of hydroxyl groups and incorporating other functionalities on the surface of GQDs, such as carboxyl groups. These groups enable electrostatic interactions with positively charged molecules, such as DOX, which contains amino groups [217], and cisplatin (CDDP), which in aqueous solutions forms positively charged dechlorinated species [219]. Additionally, doping generally increases the adsorption energy of drugs onto GQDs, making the formation of complexes thermodynamically favorable [149,220]. On the other hand, the presence of carboxyl groups on the surface of the GQDs facilitated the covalent bonding of CDDP [221] and cytarabine (Cyt) [222], while in other cases, the drugs were covalently linked with polyethylene glycol (PEG) or cysteamine [115,223,224]. These covalent bonds significantly improve control over drug loading, reduce potential leakage, and mitigate unwanted side effects [203].

The incorporation of nucleic acids into GQDs occurs mainly due to the GQDs’ unsaturated polycyclic structure consisting of hexatomic rings of carbon atoms and the nitrogenous bases of the nucleic acids, which facilitates π-π stacking without influencing the stability of DNA [225,226,227]. Unlike drugs, which require a large number of carboxyl or hydroxyl groups in GQDs for efficient loading, reducing the oxidation state and functionalizing GQDs with cationic molecules like polyethylenimine (PEI) promotes their electrostatic interaction with nucleic acids, which are negatively charged [31,214].

In terms of release, complexes can act in response to the acidic tumor microenvironment, both intracellular and extracellular [218,221,228,229]. Under these pH conditions, the H⁺ ions present in the medium tend to strengthen hydrogen bond interactions with the functional groups -COOH, -OH, and -NH2 present on GQDs, as well as with the -OH and -NH2 groups found in drugs like (DOX) [230]. This mechanism has been observed to disrupt the stabilization of loaded drugs and nucleic acids within GQDs, weakening the interactions between the components and facilitating its release. Similarly, low pH conditions promote the release of CDDP by affecting electrostatic interactions through the protonation of functional groups on GQDs, neutralizing their charge [219]. Moreover, for drugs covalently bonded to GQDs, such as Cyt or CDDP, the acidic environment triggers hydrolysis, leading to the ionization of -COOH groups to -COO^−^, and subsequently to cumulative release into the intracellular environment [222].

### 3.3. Biocompatibility and Toxicity Considerations

Comprehensive research into the biocompatibility and toxicity of nanomaterials is essential for understanding the potential risks and ensuring their safe application in biomedicine [231,232]. Biocompatibility evaluates how well a material interacts with biological systems without eliciting adverse effects; toxicity, on the other hand, refers to the overall harmful effects a substance may exert on living organisms, while cytotoxicity specifically pertains to the detrimental effects on cells [233]. Unlike common semiconductor quantum dots, which are primarily composed of materials like cadmium telluride, cadmium sulfide, cadmium selenide, and indium arsenide [195], the carbon-centric composition of GQDs renders them highly biocompatible and relatively low in toxic potential, GQDs are predominantly based on carbon, which is essential for their safe use. However, their interaction with biological systems, including potential adverse effects, is determined by their size, shape, and chemistry [39]. These interactions can influence cellular functions and processes, such as cell adhesion, proliferation, differentiation, and gene expression [234,235]. Additionally, dosage, exposure time, and aggregation state further determine the toxicity and biocompatibility of GQDs [39]. GQDs acquired through bottom-up methods or sourced from natural carbon demonstrate remarkable biocompatibility and minimal toxicity and cytotoxicity, surpassing those from top-down approaches [236,237]. Together, these elements are critical in ensuring the safety, efficacy, and bioavailability of the therapeutic agents delivered by GQDs [238].

Over the past decade, extensive research has been conducted on the potential toxicity of graphene-based materials, including GQDs [239,240]. However, different studies show varying levels of cytotoxicity [113,241,242]; this is due to slight variations in the properties of GQDs, which can significantly influence their interaction with the cell membrane, proteins, and DNA, resulting in differences among some studies and complicating the understanding of their biological activity. The size of GQDs is crucial in determining their cytotoxic effects. Both small and large GQDs can enter cells through endocytosis and phagocytosis, potentially causing general cytoskeletal injury and mitochondrial dysfunction, but they exhibit cytotoxicity through different mechanisms [240,243]. Smaller GQDs (<10 nm) can penetrate cell membranes efficiently, potentially causing significant intracellular damage. In contrast, larger GQDs, although less likely to penetrate cells deeply, can disrupt cell membranes by forming pores or extracting lipids, leading to increased cytotoxicity over smaller sizes [244,245].

Surface chemistry is another critical factor governing toxicity. Different functional groups, their position, and their abundance on the surface of GQDs lead to different cytotoxicity traits [39,246,247]. In this sense, studies have reported that some GQDs can induce oxidative DNA damage, specifically causing point mutations, and different levels of autophagy induction that suggest different cellular damage mechanisms [248,249,250]. For instance, amine and amide modifications show no cytotoxicity up to 200 μg/mL [251,252]. Additionally, GQDs with oxygen groups are predictably less toxic than more pristine GQDs, as hydrophilic materials tend to exhibit lower toxicity. In vitro and in vivo experiments demonstrated that GQDs have low toxicity due to their high oxygen content, enhancing solubility and stability, facilitating interactions with biological molecules, and preventing aggregation, which are crucial for their safe use [38]. In this sense, while larger GO sheets show significant toxicity, GQDs are comparatively less toxic [240,253,254]. However, the exact relationship between GQDs’ biocompatibility and oxygen content depends on the specific oxygen-containing groups present. In particular, hydroxylated GQDs were found to be the most cytotoxic, causing significant cell death at concentrations under 100 μg/mL. This effect is thought to be due to increased production of ROS, although the cytotoxicity of functionalized GQDs cannot be solely associated with their ability to produce ROS [245,255,256]. In contrast, carboxylic-modified GQDs showed no significant cytotoxicity, maintaining cell viability above 80% even at concentrations of 500 μg/mL in normal cells [257]. On the other hand, heteroatom-doped GQDs are generally more biocompatible than undoped GQDs, often maintaining high cell viability at the same or higher concentrations [39]. Specifically, nitrogen-doped GQDs are widely studied as a potential cancer treatment due to their high biocompatibility [258,259]. Additionally, GQDs co-doped with nitrogen and boron, sulfur, phosphorus, or neodymium also show positive interaction with biological systems [260,261,262,263,264,265]. Lastly, similar to other nanoparticles, strategies like PEGylation aim to modify the surface of GQDs and enhance their biocompatibility by reducing the generation of ROS, improving biodistribution, and enhancing clearance, even at high concentrations [38,254,266].

GQDs generally show minimal organ accumulation and are rapidly cleared through the kidneys, demonstrating low toxicity even with multiple doses in mouse models [38,249]. Studies have shown that GQDs can be broken down by human enzymes myeloperoxidase and eosinophil peroxidase within a few hours, with noticeable degradation observed after several days of treatment [267]. However, consistent with the in vitro analysis, the impact of surface modifications on biological safety must be considered. For instance, carboxylated GQDs showed very low toxicity and organ damage, as confirmed by blood counts and histological analysis, even after several days; in contrast, aminated GQDs were found to be more toxic [257,268,269]. Additionally, it is necessary to analyze the biosafety of purportedly low-toxic GQDs to ensure an impartial assessment, as some GQDs have exhibited developmental toxicity and genotoxicity in zebrafish models, as well as other forms of cell death related to neurotoxic damage [270,271].

Given the critical role of the immune system, it is of great significance to investigate the response of immune-related cells to the exposure of GQDs. Fasbender et al. discovered that in a leukocyte population that retained 90% viability after exposure to 500 μg/mL, the uptake rate via a diffusion-driven mechanism was notably higher in granulocytes and monocytes than in lymphocytes. Within the lymphoid subgroups, a significant retention was observed in CD34+ cells, NK cells, and B cells compared to cytotoxic T cells and helper T cells [272]. These variations in uptake rate could be attributed to differences in cell size and membrane permeability. In a related study, GQDs induced apoptosis and inflammatory responses in human macrophages, accompanied by expression of characteristic response factors such as interleukin-8 or tumor necrosis factor, depending on the concentration [273].

Future research directions include exploring the long-term effects of GQDs, understanding their interaction mechanisms at the molecular level, and developing safer GQD-based materials for clinical applications. Extensive research is required to optimize these factors and enhance the biocompatibility of GQDs, thereby improving their potential for drug and gene delivery in cancer treatment. Despite these challenges, ongoing research explores innovative approaches to overcome the hurdles and facilitate the clinical application of GQDs [274,275].

## 4. Applications and Novel Strategies in Cancer Treatment

### 4.1. Drug Delivery Using GQDs

GQDs have shown promise as standalone chemotherapy agents due to their ability to enter the cell nucleus and interfere with DNA activity alongside other advantageous properties stemming from their small size and aromatic ring structure, disrupting cancer cell growth [276,277]. Recent advancements include modifying GQDs with nucleus-targeting ligands to enhance efficacy directly disrupting the cancer cell nucleus [242]. Furthermore, GQDs have been investigated for their role in altering membrane permeability through interactions with lipid rafts, allowing fast translocation of drugs across lipid membranes depending on phospholipid density [278,279,280].

GQDs not only show promise as therapeutic agents but also excel as versatile platforms for drug delivery. Their high surface area and tunable surface chemistry enable precise control over the concentration of oxygen-containing groups and other functionalities [28,40,281,282], enhancing drug-loading capacity of a wide range of organic and inorganic compounds [230,283], and enabling controlled drug release [22,284]. Additionally, their planar structure allows them to pack drugs via both faces and edges and intercalate small molecules between the graphene layers, further maximizing drug-loading capacity [113]. Poorly soluble drugs and complex macromolecules have been dispersed in GQD nanosystems, enhancing drug stability and solubility, ultimately improving drug bioavailability, minimizing side effects and improving the therapeutic index of drugs [34,285,286] and overall efficacy of chemotherapy [223,287].

Incorporating functional groups and linker molecules on the surface of GQDs allows the binding of the complex components through electrostatic interactions and covalent bonds. This prevents the nonspecific release of the drug, improves cellular uptake, promotes intracellular accumulation, and reduces therapeutic doses by up to 10 times compared to the drug-free versions [203,217,241]. Additionally, doping modulates GQDs’ properties, increasing drug absorption and fluorescence [28,220]. Other promising modifications include incorporating proteins, such as serum albumins, which increase the bioavailability and prolong the half-life of the loaded drug [193,288]. Integration of polymers, like PEI and PEG, improves solubility, dispersion, and biocompatibility of GQDs in aqueous media, in addition to increasing the drug-loading capacity and reducing the interaction with macrophages or other non-specific agents in the blood [224,228,289,290].

While some GQDs have the ability to induce oxidative stress [291], others exhibit antioxidant properties [215]. This duality arises from their unique and variable properties attributed to their intrinsic nature [243], doping [292], or functionalization with photosensitizing molecules [179]. For example, researchers have enhanced the oxidative damage capabilities of GQDs by co-doping them with nitrogen and phosphorus, enabling them to efficiently convert hydrogen peroxide into hydroxyl radicals within cancer cells, a process driven by a synergistic electron effect [261]. Additionally, the radical scavenging abilities of GQDs, as previously discussed in their capacity to neutralize free radicals through hydrogen donation, are crucial for mitigating oxidative stress. This stress is closely associated with tumor growth, metastasis, and drug resistance [273]. By effectively reducing oxidative stress, these antioxidant properties of GQDs could potentially enhance the effectiveness of drug treatments in cancer therapy [274,275,276]. Moreover, GQDs exhibit the ability to damage chemotherapy-resistant cells, such as MCF-7/ADR [277]. GQDs interact with cytosine-rich regions of promoters of drug transporter genes like P-glycoprotein, multidrug resistance protein 1 (MRP1), and breast cancer resistance protein (ABCG2), thereby negatively regulating the expression of multiple multidrug-resistant genes, allowing drugs to remain within the intracellular medium and exert their therapeutic activity [278].

The functionalization of GQDs with ligands targeting overexpressed receptors on cancer cells has been a strategy for targeted drug delivery [293]. For instance, gemcitabine has been delivered through GQDs functionalized with hyaluronic acid targeting the CD-44 receptor in pancreatic cancer cells [288]. Similarly, DOX has been administered to lung cancer cells through functionalization with biotin [294]. This has significantly increased the cytotoxicity of the drug compared to the non-functionalized complex and the drug in its free state. For example, one study demonstrated that CDDP administered to breast cancer cells via GQDs functionalized with an antibody fragment (scFvB10) targeting the epidermal growth factor increased treatment efficacy [219]. Another study showed that DOX delivered through GQDs functionalized with an RGD peptide had favorable treatment results [283,295]. Moreover, research involving folic acid targeting the folate receptor has shown that targeted delivery to breast cancer cells reduces the side effects of tamoxifen in normal cells [224] and can reduce tumor size by up to 99.68% in mouse models when mitoxantrone is administered [218]. Furthermore, the targeting capability of GQDs, combined with their intrinsic fluorescence or additional fluorescent molecules, has enabled their application as theranostic agents for diagnosing and monitoring cancer treatment [290,296,297].

The combination of anticancer drugs prevents drug resistance in oncological therapy, increases efficacy at lower doses, and reduces side effects [298]. This was demonstrated in a study where the large surface area of GQDs was utilized to simultaneously load DOX and CDDP, achieving a reduction of up to 10 times in tumor weight compared to the control group without causing alterations in blood chemistry [216]. Similarly, GQDs also combine their drug administration ability with their capabilities to act as photosensitizers when exposed to light of specific wavelengths [265]. This exposure produces oxidative stress in PDT and hyperthermia in PTT, demonstrating a synergistic therapeutic effect [179,299]. For instance, a study improved the therapeutic effect in pancreatic cancer by combining chemotherapy with DOX, gene therapy, and PTT, increasing the number of cells in the apoptosis state to 74% [300]. Another study demonstrated that silver nanoparticles decorated with GQDs loaded with DOX reduced cell viability from 75% to 68% after irradiation due to the enhanced effect of PDT [289]. Similarly, another research effort used a complex of nitrogen-doped GQDs (N-GQDs) as a sensitizing agent for PDT, DOX as chemotherapy, and 3-Aminopropyltriethoxysilane (APTES) as a charge reversal agent to facilitate drug delivery to the nucleus, significantly enhancing cancer therapy [301]. Alternatively, PTT using GQDs loaded with IR-780, a theranostic agent capable of increasing local temperature and inducing hyperthermia, contributed to the effectiveness of the treatment and nearly eliminated the tumor in a mouse model [302]. A theranostic system of GQDs released its aptamer to hybridize with a cancer biomarker (miRNA-155), allowing the differentiation from healthy cells through fluorescence. This system applied different wavelengths to increase the temperature in the PTT and activate the loaded porphyrins to produce singlet oxygen from molecular oxygen in alveolar carcinoma and breast cancer cell lines, demonstrating that the combination of both therapies is capable of reducing cell viability up to 14% [297].

In recent studies, GQDs act as a supporting element, combining their properties with those of more complex drug delivery systems (DDS) in cancer therapy. Firstly, combining GQDs with delivery systems constituted by polymers, such as chitosan, improves the biocompatibility and intracellular accumulation of the drug [303]. Carboxymethylcellulose provides greater mechanical strength [304], and poly(lactic-co-glycolic) acid (PLGA) helps maintain the complex’s stability by protecting it from oxygen and water [193]. Additionally, GQDs have endowed other nanoparticles with pH-responsive drug release, light sensitivity, monitoring abilities, and fluorescence imaging, fostering the evolution of more intricate strategies [305,306,307,308]. For example, to increase biocompatibility, wrapping the complexes with a red blood cell membrane prevents their interaction with macrophages and extends their circulation time [309]. Similarly, applying GQDs in a dual DDS carrying two distinct therapeutic agents improves treatment effectiveness by enhancing drug loading and release, combating drug resistance, and decreasing toxicity toward healthy cells [197]. The therapeutic effect was improved by subjecting magnetic hollow nanoparticles decorated with DOX-loaded GQDs to a magnetic field, providing mechanical stress and chemotherapeutic damage to synergistically eliminate cancer cells [310].

GQDs used in drug delivery have been predominantly synthesized from precursors such as GO [283,288,311], graphite, [216,283,312], acidic compounds like L-glutamic acid and citric acid, and their respective salts [113,224,307], polymers like polythiophene [313], sugars like glucosamine [241], other nanomaterials such as multi-walled carbon nanotubes [294], and materials like CX-72 carbon black [219].

Although GQDs are a promising alternative for drug delivery, more detailed and comprehensive investigations on their drug-loading and -release properties are necessary [35,284]. Additionally, exploring doping and functionalization mechanisms with previously untested elements and molecules to modify their properties based on their interaction with drugs, cancer cells, and the organism is crucial [31,104,314]. Combining GQDs with other drug delivery systems to develop more effective platforms in cancer therapy could lead to the first clinical trials and products in this field [315]. Moreover, the interaction between biomaterials like GQDs and immune cells plays a vital role in designing innovative and effective drug delivery systems capable of eliciting immune responses [316]. Zhang et al. synthesized a stable hybrid GQD-PEG photosensitizer with photodynamic efficiency and immunostimulatory properties, inducing tumor cell death through ROS generation upon irradiation, followed by immune response activation via inflammatory cytokine secretion, ultimately enhancing therapeutic efficacy [317].

Table 2 summarizes the functionalization strategies and pharmacological interactions of GQDs, highlighting data from both in vivo and in vitro experiments.

### 4.2. Gene Delivery and Gene Therapy with GQDs

While most research focuses on drug delivery, GQDs display encouraging opportunities in transporting various substances beyond pharmaceuticals, including DNA and peptides [34]. Cancer gene therapy, in particular, significantly relies on efficiently delivering nucleic acids (DNA or RNA) to an individual’s tumor to manipulate the genetic material or modulate gene expression [10]. However, the therapeutic potential of naked nucleic acids is limited by several barriers, such as size and charge constraints, degradation by extracellular nucleases, and entrapment within endosomal compartments. Hence, developing efficient delivery systems is crucial for gene therapy [319]. As such, utilizing the unique properties of GQDs to design specifically tailored platforms becomes particularly advantageous.

GQDs have demonstrated their versatility in facilitating gene expression by effectively delivering plasmid DNA (pDNA) and messenger RNA (mRNA). Ahn et al. demonstrated that positively charged N-doped GQDs coated with PEI could effectively deliver mRNA or pDNA into HeLa cells with low cytotoxicity [214]. The transfection efficiency with pDNA was similar to that of the positive control, while for mRNA it reached 27%, compared to the 18% achieved with Lipofectamine. Conversely, utilizing a higher molecular weight PEI for the functionalization of N-doped GQDs resulted in GQDs/mRNA complexes achieving up to 25% transfection efficiency at low doses in Huh-7 hepatocarcinoma cells, also capable of retaining considerable activity in a shear tolerance test [320]. However, these complexes were less effective and more toxic than the reference lipid nanoparticles. Notably, while the cationic charge intended to facilitate cellular uptake can enhance interaction with cell membranes, it can also lead to cytotoxic effects [321,322].

Other strategies have enhanced transfection efficiency [323]. As previously mentioned, modifying COOH-functionalized GQDs with PEI and an antibody targeted to EGFR enhanced the performance of a multifunctional system carrying a pDNA with a reporter gene and DOX in colon cancer cells [290]. When pDNA encoding the firefly luciferase gene was complexed with a positively-charged chimeric peptide (MPG-2H1 peptide) specifically designed to disrupt the endosomal membrane and containing a nuclear localization signal (NLS), subsequently attached to GQDs by electrostatic interactions, the complexes were capable of overcoming cellular trafficking barriers and showed a five-fold increase in transfection efficiency in HEK293t cells compared to the MPG-2H1 peptide/pDNA complex alone [324]. This was the first report on gene delivery using GQDs and could apply to various types of gene therapy, including potentially for cancer. Interestingly, they obtained two different sizes of GQDs with two different green and red emissions from the synthesis, but they did not separate the two populations before forming the complexes.

In addition, GQDs are a promising solution for enabling gene inhibition by efficiently delivering small interfering RNA (siRNA) and microRNA (miRNA). Even though the EphA2-siRNA loaded onto Eudragit-coated N-GQDs targeting the EphA2 receptor overexpressed in many solid tumors did not significantly reduce the viability of A549 lung tumor cells, a higher cell death rate was observed, likely due to the combined impact of the DNA effects from the GQDs and the effects of the siRNA [325]. A more efficient approach was designed by Yang et al., a novel nanocarrier consisting of N-doped GQDs functionalized with a biodegradable cationic polyester vector (BCPVs) and co-loaded with both DOX and a K-ras siRNA [300]. The resulting nanocomplexes exhibited high stability and demonstrated effective downregulation of K-ras expression, leading to significant inhibition of proliferation in MiaPaCa-2 pancreatic cells. The authors showed that BCPV improved the stability, biocompatibility, and cellular uptake of the loaded siRNA, aiding its escape from endosomes into the cytosol [326]. Additionally, the downregulation of mutant K-ras expression enhanced cell apoptosis, inhibited cell metastasis and colony formation, and is related to reducing drug resistance [327]. NIR-triggered photothermal effects further enhanced the synergistic anticancer activity of DOX and K-ras siRNA by improving cellular availability. In a separate study, nitrogen (N) and neodymium (Nd) were employed as doping ions to produce positively charged GQDs [264]. The NGQD/siRNA and Nd-NGQD/siRNA complexes effectively inhibited the expression of EGFR and K-ras proteins in HeLa cells by approximately 31-51%, demonstrating comparable efficacy to the Lipofectamine positive control. Furthermore, the addition of Nd enhanced the complexes’ NIR imaging capabilities.

Dong et al. designed a more intricate nanosystem by grafting PEG and PLA onto N-doped GQDs [328]. This nanosystem combined inhibitory probes (IP) and antisense oligodeoxynucleotide (ASODN) targeting miRNA-21 and survivin to enhance gene therapeutic efficacy. As a result, cell viability decreased to 68%, contrasting with GQDs loaded solely with IP (82% viability) or ASODN (76% viability). Moreover, cells transfected with the combination exhibited a significantly higher apoptotic ratio than those transfected with ASODN alone. This and other systems are also useful for detecting the presence of intracellular cancer-related miRNA [297].

Finally, drawing from the referenced studies, GQDs used for gene delivery in cancer have been synthesized from various sources, including natural compounds such as citric acid and glucosamine hydrochloride and other materials like graphite powder. Future perspectives include exploring green synthesis methods using natural carbon sources aiming for more sustainable and environmentally friendly production processes.

Although GQDs are not as extensively advanced as other carbon agents like GO [36,329], they hold promise as next-generation therapeutics for gene delivery in cancer therapy. Overcoming specific barriers, such as optimizing surface functionalization, endosomal escape, nuclear targeting, gene binding/release, biodegradability, immunogenicity, and in vivo tracking, is essential for their full development as gene therapy vectors. Similarly, for drug delivery, no clinical trials or approved products are currently available. Existing imaging strategies should be implemented towards modulating cancer-related genes, while other complex combinatorial therapies, such as those demonstrated in recent studies involving monocytes decorated with N-doped GQDs for miR223 delivery to macrophages within atherosclerotic plaques, show promise for targeted gene expression regulation and inflammation reduction, with potential implications for cancer therapy [330].

**Figure 3:**
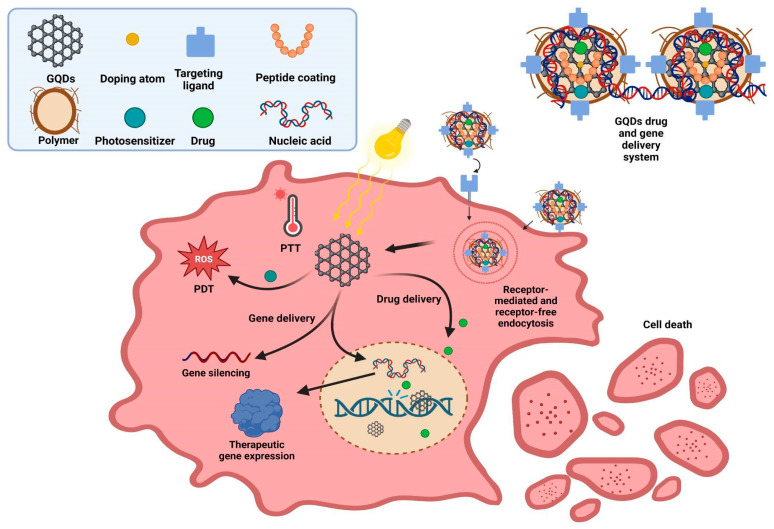
Drug and gene delivery for cancer therapy. The figure illustrates the entry of a multifunctional GQD-based drug and gene delivery system through receptor-mediated and non-receptor-mediated endocytosis. This system inherently possesses photothermal properties and is modified with peptides and polymers that enhance its bioavailability, biocompatibility, half-life, and physicochemical properties. The figure also shows a targeting ligand molecule that guides the complex specifically toward cancer cells. Additionally, this system is loaded with a photosensitizer agent that induces oxidative stress as part of photodynamic therapy, a drug that exerts its biological activity by causing DNA damage, and nucleic acids that express therapeutic proteins or silence damage-causing genes. All these components share the common goal of inducing tumor cell death. Figure was created using Biorender.com (accessed on 1 June 2024).

Figure 3 summarizes the different drug and gene delivery strategies based on GQDs systems used to eliminate cancer cells.

## 5. Conclusions and Future Perspectives

GQDs represent a promising frontier in cancer therapy due to their unique properties and versatile applications. These nanomaterials possess several advantageous characteristics, such as biocompatibility, non-toxicity, and high solubility. Their large surface area and flexibility in functional group manipulation allow for easy attachment of drugs and ligands, enhancing the stability and solubility of anticancer agents and making them effective for drug and gene-targeted delivery. GQDs hold significant promise in addressing research gaps in cancer therapy by offering synergistic treatment modalities and enhanced therapeutic efficacy, reducing toxicity compared to traditional approaches. Their ability to induce DNA damage in cancer cells, ROS generation, and enhancement of PDT and PTT contributes to tumor suppression. Optimizing the size and shape of GQDs can influence cellular uptake and gene delivery efficiency, while stimuli-responsive GQDs offer controlled gene release at specific sites. Hybrid systems combining GQDs with other materials further optimize delivery mechanisms, enhancing precision. Moreover, the interaction between GQDs and immune cells demonstrates promising potential for advancing cancer immunotherapy, enhancing therapeutic efficacy through tumor cell death and immune response activation.

However, despite these promising advancements, realizing the full biomedical utility of GQDs requires addressing several challenges and limitations in their application for cancer therapy. Ensuring biocompatibility and minimizing toxicity are primary concerns, necessitating precise synthesis techniques and surface functionalization methods. The synthesis of GQDs from natural carbon sources like biomass and organic waste highlights their potential for sustainable and scalable manufacturing. Top-down methods, such as electrochemical and liquid-phase exfoliation, are advantageous due to their ability to produce GQDs in large quantities and with relatively straightforward procedures. These methods, however, can yield mixtures of GQDs and larger graphene particles, posing a challenge in achieving uniform size and quality. Bottom-up methods, like pyrolysis and catalytic solution chemistry, offer higher precision in GQD morphology but can be more complex and resource-intensive. Refining synthesis protocols to ensure consistent, high-quality GQDs for clinical use remains a significant challenge. The methods used impact the physical, chemical, and biological properties of GQDs, influencing their effectiveness as therapeutic agents. Therapeutic efficacy also depends on the efficiency of drug-loading and controlled release systems, as well as strategies for ensuring stable dispersion and precise targeting. Achieving precise control over GQDs’ size, surface chemistry, and crystalline structure is difficult but crucial for their application in drug and gene delivery.

These findings underscore the importance of leveraging GQDs in future clinical trials to develop effective cancer therapies. While research on the in vitro and in vivo toxicity of GQDs is expanding, comprehensive studies are still needed to fully understand their safety profile. Despite the promising features and versatile applications of GQDs, the adoption of this technology in clinical settings has been slow. According to the National Institute of Health (NIH) database, as of today, only one clinical trial involves using GQDs in conjunction with Si nanowires as an early diagnostic tool for acute myocardial infarction (NCT04390490). This underscores the need for further research before these materials can be applied to biomedical applications. Lastly, to advance cancer treatment strategies using GQDs, further exploration, interdisciplinary research, and collaboration are encouraged. Researchers have highlighted the importance of GQDs in drug delivery, bioimaging, and targeted therapy, emphasizing the need for continued investigation into their future applications. Collaborative efforts between nanotechnology, medicine, and materials science experts can lead to innovative approaches in utilizing GQDs for cancer therapy, ultimately improving patient treatment outcomes.

## Figures and Tables

**Table 1:** Carbon sources and methods used for the synthesis of GQDs, their functionalization, average size and thickness, and applications.

Carbon Source	Methods	Functionalization	Average Size/Thickness	Application	Reference
Rice grains	Pyrolysis	-OH, C-H, C-O-C, C=O	2.0–6.5 nm/–	Biomarkers for cell imaging	[105]
Carbon black, nitric acid	Liquid-phase exfoliation	C-N, N-H, -NH-CO-	1.0–4.0 nm /<0.8 nm	Cellular imaging, drug delivery	[106]
Mango leaves	Microwave	-OH, C=C, C-C	2.0–8.0 nm/–	Bioimaging, cytoplasm labeling	[107]
Graphene oxide	Hydrothermal	C-N	1.84 nm/–	Biological imaging	[108]
Cotton cellulose	Hydrothermal	O-C=O, C-OH, C=O, C=C/C-C	3.0–3.5 nm /0.5–0.8 nm	In vitro imaging	[109]
Rice husk	Hydrothermal	–	3.0–6.0 nm /0.8–1.6 nm	Cell imaging	[110]
Graphene oxide, Ammonium hydroxide	Hydrothermal	C-O, C-C, C=C, C-N, N-H	4.0–6.0 nm/–	Cells imaging, Osteogenic differentiation	[111]
Carbon black	Liquid-phase exfoliation	–	–/1.0 nm	Tumor cell labeling and photothermal therapy	[112]
Citric acid, urea	Hydrothermal	O-H, N-H, C=O, C-N	2.0–5.0 nm/–	Methotrexate-loaded drug delivery system	[113]
L-glutathione (Glycine, cysteine, and glutamic acid)	Hydrothermal	C=C, C-O-C, C=O		Cancer theranostics	[114]
Cow milk	Microwave	C=C, C-O, C-N, C=N, O-C=O	5.0 nm/–	Cancer theranostic system	[115]

**Table 2:** Functionalization and pharmacological interaction characteristics of graphene quantum dots in in vivo and in vitro assays.

Functionalization/Doping	Drug	Size of Complex	Interaction (GQDs-Drug)	Characteristics	Assays				Reference
					In Vitro		In Vivo		
					Cell Line	Evaluated Concentrations	Animal Model	Evaluated Concentrations	
-	DOX	GQD-DOX: 99–107 nm	π-π	Rapid nuclear uptake Biocompatibility	MCF-7 WI-38	3.125 up to 200 µg/mL of GQDs-DOX	Albino Wistar rats	5 up to 20 mg/kg of GQDs	[217]
N	DOX	-	π-π	Increased biocompatibility	MCF-7 HeLa HEK-293	0.00625 up to 1.6 µg/mL of DOX	-	-	[241]
N, S and Immobilized on MSN	DOX	MSN-GQD: 49.5 nm	Non-covalent bond	Greater internalization Biocompatibility	HeLa MSU-1.1	20 up to 400 µg/mL of MSN-GQDs	-	-	[307]
-COOH and Cu +2	DOX	-	π-π	Increased cytotoxicity	MCF-7/ADR	50 up to 250 µg/mL of GQDs	-	-	[311]
N	MTX	-	π-π	Increased biocompatibility	MCF-7	0 up to 4 µM of MTX-GQD	-	-	[113]
BTN	DOX	-	π-π	Increased targeting Increased biocompatibility	A549	3.75 up to 15 µg/mL of GQDs-BTN-DOX	-	-	[294]
scFvB10	CDDP	-	Electrostatic interactions	Increased targeting	MDA-MB-231	5 up to 75 µg/mL of CDDP	-	-	[219]
FA and mPEG2000	TMX	TMX-FPG: 294.7 nm.	π-π	Increased targeting	MCF-7 HDF	1 up to 200 µg/mL of TMX-FPG	-	-	[224]
HA	HSA-encapsulated gemcitabine	HSA-NPs: 150 nm	(CO–NH)	Increased targeting Increased drug half-life	Panc-1	5 up to 400 µg/mL of HSA-NPs	-	-	[288]
RGD	DOX	-	π-π Hydrophobic interactions.	Increased targeting Increased toxicity	U251	0 up to 200 µg/mL of RGD-GQDs-DOX	-	-	[283]
PEG and FA	MTN	FA-PEG-cGQDs: 4–10 nm	π-π Hydrogen bonds	Increased targeting Increased biocompatibility	HeLa	0.05 up to 1 μg/mL of MTN	Athymic nude mice	2.5 mg/kg of MTN.	[218]
FA	IR780	-	π-π	Increased targeting Combination with photothermal therapy	HeLa	0 up to 30 µg/mL of FA-GQDs- IR780	Balb/c nude mice	2 mg/kg of FA-GQDs- IR780	[302]
GE11	CDDP DOX	GE11-GQD:14.23 nm	π-π	Increased targeting Combination of two pharmacological therapies	CNE-2	0 up to 100 µg/mL of GE11-GQD	Balb/c nude mice	2 mg/kg of DOX and CDDP	[216]
-	B-Lap	-	π-π	Greater antitumor effect compared to the free drug High biocompatibility Rapid release of the drug in aqueous medium	LO2 HeLa PC-12 MCF-7	5 up to 10 uM of B-Lap	-	-	[312]
Cy5.5	DOX	DOX-GQDs-P-CY: 15.6 nm	π-π	High therapeutic activity in vitro and in vivo Theranostic and monitoring agent	4T1	0 up to 4 µg/mL of DOX-GQDs-P-CY	Orthotopic mice model	-	[296]
BM and PEG	Porphyrin	-	-	Detection of cancer-associated miRNAs Efficient photothermal conversion High production of singlet oxygen	A549	10 up to 200 µg/mL of GQD-PEG-P	-	-	[297]
PEI and EGFR	DOX	GQDs-DOX: 40–80 nm	π-π	Tumor suppression capacity Longer-sustained inhibition capacity Less toxicity than DOX	HCT-116	<5 μM of GQDs-DOX	Nude mice	200 μL of GQDs-DOX	[290]
hMSN, PEG and VEGF	DOX	GQDs-hMSN-PEG-VEGF-DOX and GQDs-hMSN-PEG-PEG-DOX: 100–150 nm	-	Biocompatibility High DOX loading efficiency Structural stability Enhanced DOX delivery High specificity	MCF-7 L929	0–10 μg/mL of GQDs-hMSN-PEG-VEGF-DOX and GQDs-hMSN-PEG-PEG-DOX	BALB/c nude mice	5 mg/kg of GQD-hMSN	[313]
HA	DOX	GQD-HA: 20 nm		Increased targeting Increased toxicity	A549	0.5–5 μg/mL of GQD-HA-DOX	BALB/c mice	10 mg/kg of GQD-HA	[318]

Note: DOX: Doxorubicin; MCF-7: Human breast cancer; WI-38: Human fetal lung fibroblasts; N: Nitrogen; HeLa: Human cervical cancer; HEK-293: Human embryonic kidney; S: Sulfur; MSN: Mesoporous silica nanoparticles; MSU-1.1: Human fibroblasts; Cu^+2^: Copper II ion; MCF-7/ADR: Human breast cancer with doxorubicin resistance; MTX: Methotrexate; BTN: Biotin; A549: Human alveolar carcinoma cells; scFvB10: Single chain variable fragment antibody; CDDP: Cisplatin; MDA-MB-231: Human breast adenocarcinoma; FA: Folic acid; mPEG2000: Methoxypolyethylene glycol; TMX: Tamoxifen; TMX-FPG: mPEG and FA-functionalized GQDs loaded with TMX; HDF: Human dermal fibroblasts; HA: Hyaluronic acid; HSA-NPs: Hyaluronic acid conjugated graphene quantum dots and human serum albumin nanoparticles; Panc-1: Human pancreatic epithelioid carcinoma; RGD: Arginine, glycine, aspartic acid; U251: Human glioma cells; Ag: Silver; PEG: Polyethylene glycol; DU145: Human prostate cancer; NH2-PEG-NH2: Aminated polyethylene glycol; MTN: Mitoxantrone; FA-PEG-cGQDs: IR780: Theranostic agent for fluorescence imaging and photothermal therapy; GE11: Peptide targeting epidermal growth factor; CNE-2: Human nasopharyngeal carcinoma; B-Lap: B-Lapachone; LO2: Human liver cells; PC-12: Mouse neural cells; Cy5.5: Cyanine-5.5; 4T1: Mouse T cells; BM: Aptamer for miRNA 155 detection; PEI: Polyethylenimine; EGFR: Epidermal growth factor receptor; HCT-116: Human colorectal cancer cells; hMSN: hollow mesoporous silica nanoparticles; L-929: Mouse fibroblasts.
